# A ferroptosis-related gene signature for graft loss prediction following renal allograft

**DOI:** 10.1080/21655979.2021.1953310

**Published:** 2021-08-01

**Authors:** Zhenlei Fan, Tao Liu, Hanfei Huang, Jie Lin, Zhong Zeng

**Affiliations:** Organ Transplantation Center, The First Affiliated Hospital of Kunming Medical University, Kunming, Yunnan, P. R. China

**Keywords:** Ferroptosis, renal allograft, prognosis, gene signature, nomogram

## Abstract

Allogeneic kidney transplantation (renal allograft) is the most effective treatment for advanced kidney disease. Previous studies have indicated that ferroptosis participates in the progression of acute kidney injury and renal transplant failure. However, few studies have evaluated the prognostic value of ferroptosis on renal transplantation outcomes. In this study, a total of 22 differentially expressed ferroptosis-related genes (DFGs) were identified, which were mainly enriched in infection-related pathways. Next, a ferroptosis-related gene signature, including GA-binding protein transcription factor subunit beta 1 (GABPB1), cyclin-dependent kinase inhibitor 1A (CDKN1A), Toll-like receptor 4 (TLR4), C-X-C motif chemokine ligand 2 (CXCL2), caveolin 1 (CAV1), and ribonucleotide reductase subunit M2 (RRM2), was constructed to predict graft loss following renal allograft. Moreover, receiver operating characteristic (ROC) curves (area under the ROC curve [AUC] > 0.8) demonstrated the accuracy of the gene signature and univariate Cox analysis suggested that the gene signature could play an independent role in graft loss (p < 0.05). Furthermore, the nomogram and calibration plots also indicated the good prognostic capability of the gene signature. Finally, immune-related and cytokine signaling pathways were mostly enriched in renal allograft patients with poor outcomes. Considered together, a ferroptosis-related gene signature and nomogram based on DFGs were created to predict the 1-, 2- and 3- year graft loss probability of renal allograft patients.The gene signature could serve as a valuable biomarker for predicting graft loss, contributing to improving the outcome of allogeneic kidney transplantation.

## GRAPHICAL ABSTRACT

**Figure:**
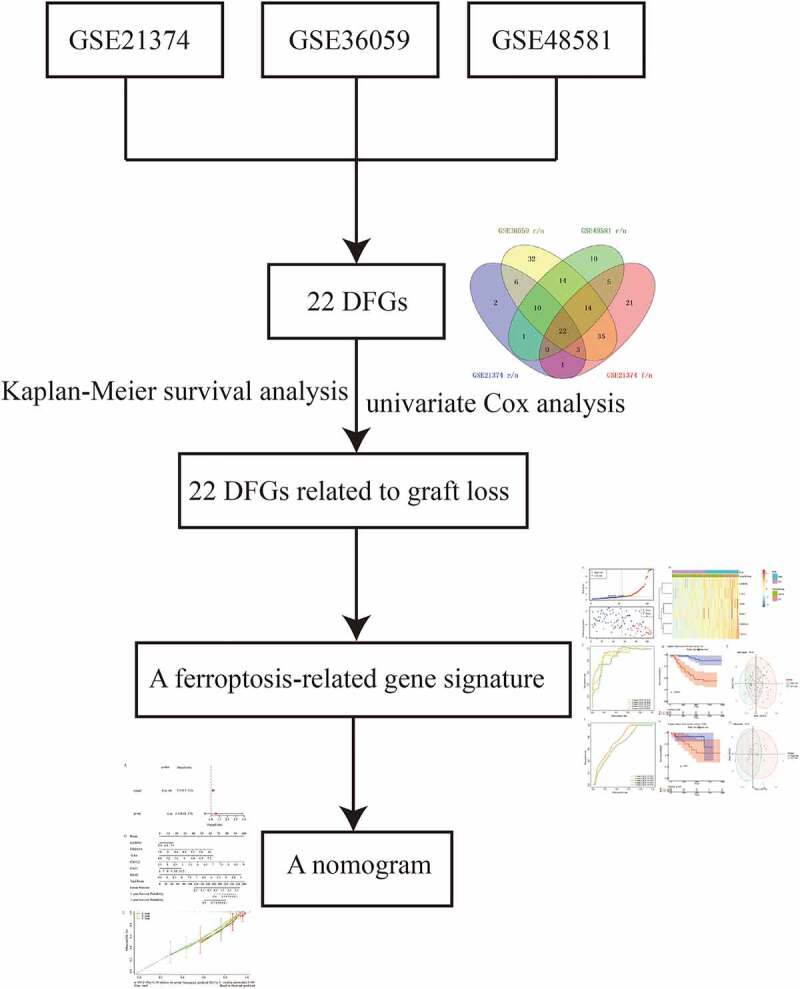

## Introduction

Allogeneic kidney transplantation (renal allograft) is the treatment for patients with end-stage renal disease and severe chronic kidney disease [1]. It has been estimated that approximately 120,000 new organ transplantations are carried out annually worldwide [2]. However, the success rate of renal allografts remains low, with only one million individuals obtaining functioning solid-organ transplants [2]. Moreover, approximately 5% of primary graft non-function occurs in the first year following kidney transplantation [3]. Hence, identifying graft loss-associated biomarkers might contribute to the treatment of graft loss and improve the efficiency of renal allografts.

It has been suggested that antibody-mediated immune rejection following renal allograft is associated with graft loss and the death of patients [3,4]. In addition, antibody-mediated immune rejection has long-term negative effects on renal allografts [5], and is a major cause of renal fibrosis [6,7]. More importantly, native kidney disease recurrence following immune rejection is the second most predominant cause of graft loss [4]. Thus, the genes involved in the rejection process might affectgraft loss after renal allografts.

Ferroptosis, a newly discovered form of cell death, is characterized by lethal accumulation of lipid peroxidation [8,9]. Recent studies have revealed that ferroptosis is associated with the occurrence and progression of many diseases, including cancer, myocardial infarction, and neurological diseases [10–13]. To date, many genes that are involved in the process of ferroptosis by changing the cellular levels of lipid peroxidation and iron have been discovered [14]. For example, glutathione peroxidases 4 (GPX4) can inhibit ferroptosis by regulating the level of lipid peroxidation [15]. Moreover, it has been found that p53 also participates in ferroptosis [16]. Ferroptosis has been suggested to play a key regulatory role in acute kidney injury [17,18]. Furthermore, inhibition of ferroptosis protected the the cells from injury in a vitro model of acute injury in renal tubular cells [19]. On the other hand, it has been demonstrated that ferroptosis can also affect renal ischemia/reperfusion (I/R) injury [20]. Nevertheless, the role of ferroptosis in antibody-mediated immune rejection following renal allograft remains unknown. Furthermore, the the potential use of ferroptosis-related genes as biomarkers for graft loss prediction needs to be investigated.

In the present study, we hypothesized that ferroptosis-related genes could play a role in antibody-mediated immune rejection following renal allograft and that they could act as biomarkers for graft loss prediction. The aim of this study was to establish a ferroptosis-related gene signature for predicting graft loss using comprehensive bioinformatic analyses. Furthermore, our goal was to increase the understanding of the roles of ferroptosis in antibody-mediated immune rejection and contribute to developing a new strategy for graft loss prediction following renal allograft.

## Methods

### Data collection

The GSE21374, GSE36059, and GSE48581 datasets, including clinical and RNA expression profiling datasets of human renal allografts, were downloaded from the Gene Expression Omnibus (GEO) database (*https://www.ncbi.nlm.nih.gov/geo/query/acc.cgi*) [21]. All tissue samples in these three datasets were from graft rejection and non-rejection patients who were identified using biopsy after renal allograft. Moreover, GSE21374 also included failed (graft loss) and non-failed information, and samples were obtained from two patient batches. One batch including 105 patients who survived over one year after surgery, collected at UIUC or University of Alberta from September 2004 to October 2007, was defined as the training set. The second batch, including 48 patients living for more than one year after surgery, collected from September 2006 to September 2007 at the University of Minnesota, was considered as the validation set. In addition, we obtained 259 ferroptosis genes from the FerrDb database (*http://www.zhounan.org/ferrdb/*). These genes were classified into driver, suppressor, and marker gene groups in the database.

### Identification of differentially expressed ferroptosis genes (DFGs)

Firstly, we selected ferroptosis-related genes from the three expression matrices. Then, the R package ‘limma’ was used to screen the differentially expressed genes between the rejection and non-rejection groups in each dataset [22]. It was then used to select the differentially expressed genes between the group in which the graft loss time was less than the median survival time and the group without graft loss and more than the median survival time in the GSE21374 dataset (adj.P.Val ≤ 0.05 was set as a criteria). The volcano plots of the DFGs were plotted using the R package ‘ggplot2’ [23]. Finally, we identified the common DFGs based on the overlapping differentially expressed genes using the R package ‘Venn diagram’ [24].

### Functional enrichment analysis

To explore the biological function of the DFGs in renal allografts, the Gene Ontology (GO) function and Kyoto Encyclopedia of Genes and Genomes (KEGG) pathway enrichment for the DFGs were analyzed using the R package ‘clusterprofiler’ R package [25]. A *P* -value < 0.05 was considered as statistically significant. In addition, the top 10 GO terms and KEGG pathways were visualized with a bubble diagram by using the R package ‘ggplot2’ [23].

### Protein-protein interaction (PPI)

To identify the functionally significant genes, we uploaded all DFGs to the STRING database (*https://string-db.org/cgi/network.pl*) to generate a PPI network. Genes that were highly interconnected with nodes and interaction scores > 0.4, were considered as hub genes. Then, we used Cytoscape to visualize the network [26].

### Screening graft loss related DFGs

Kaplan-Meier survival analysis was employed to screen graft loss-related DFGs based on their expression levels in renal allograft patients [27]. The log-rank test was used to determine the *p* value using the R package ‘survminer’ [28]. A *p* -value of < 0.05 was considered as statistically significant.

### Construction of the prognostic gene signature

A gene signature was generated to predict graft loss in renal allograft patients in the training set and then verified in the validation set. Firstly, a univariate Cox proportional hazards regression model was performed to identify candidate graft loss-related DFGs by using the R package ‘survival’ [29], and genes with a p value of < 0.05 were further confirmed using LASSO COX regression. In the LASSO analysis, the combination of independent variables can be screened and a better fit can be obtained by adding a constraint condition to the sum of the absolute values of the coefficients to reduce the dimensionality of high-dimensional data [30]. During the analysis, the R package ‘glmnet’ was used for LASSO COX analysis [31]. ‘Cox’ was set as the family parameter, and the ten-fold cross validation was used to realize the Lasso logistic regression. Moreover, genes identified in the LASSO analysis were set as covariates and included in the multivariate Cox regression analysis. Finally, the gene signature was established based on the expression values and regression coefficients.

### Evaluation of the predictive capability of the gene signature

We calculated the risk score of all samples from renal allograft patients by using the predict.coxph function [32], as follows: risk score = esum (each gene’s expression levels × corresponding coefficient)/ esum (each gene’s mean expression levels × corresponding coefficient). Patients with a risk score greater than the median value were assigned to the high-risk group, otherwise, they were assigned to the low-risk group. Then, the R package ‘survivalROC’ was used to generate 1-, 2-, 3-, 4-, 5-year receiver operating characteristic (ROC) curves to evaluate the accuracy of the gene signature in predicting graft loss in renal allograft patients [33 34]. Moreover, Kaplan-Meier analysis was performed to observe the difference in graft loss time by using the log-rank test (*p* < 0.05) [27,28]. Finally, scatter plots were used to evaluate the distance between the two groups using principal component analysis (PCA).

### Identification of independent prognostic factors of graft loss

We firstly evaluated the relationship between the risk score and graft loss. Then, the risk score and graft reaction were analyzed using univariate Cox regression to confirm the risk factors for overall survival (OS) [29].

### Construction of the predictive nomogram

We integrated the independently predictive factors and constructed a nomogram using the R package ‘rms’ to inspect the probability of 1- and 3- year graft loss in renal allograft patients in the GSE21374 dataset [34]. A calibration curve was plotted to observe the nomogram prediction probabilities against the observed graft loss rate.

### Gene set enrichment analysis (GSEA)

To explore the potential mechanisms differentiating the high- and low-risk groups, we firstly compared the differentially expressed genes between the high risk group to low risk groups in the GSE21374 dataset by using the R package ‘limma’ [22], with the screening thresholds of *p* < 0.0.5 and |log2FC | > 1. Next, all genes were ranked according to the log2FC value, and GSEA was conducted to search for GO and KEGG pathway terms for the two groups [25].

### Quantitative Real-Time-PCR Validation

To further analyze the roles of genes in ferroptosis-related gene signature, we firstly compared the expression levels of genes in ferroptosis-related gene signature in GEO database. Next, we collected 5 rejection peripheral blood samples and 5 non-rejection peripheral blood samples from the First Affiliated Hospital of Kunming Medical University. The informed consent was obtained from all participating individuals; the study was approved by the Ethics Committee at the first Affiliated Hospital of Kunming Medical University.

Peripheral blood mononuclear cells (PBMCs) were separated within 4 h of blood withdrawal using Lympholyte Cell Separation Media (CEDARLAN, Canada). Total RNAs were extracted from rejection and non-rejection PBMCs by the TRNzol-A+ Reagent (TIANGEN) based on the manufacturer’ s guidance. Next, purified RNA was reverse transcribed complementary DNA (cDNA) using the FastQuant RT Kit (TIANGEN) according to the manufacturer’s procedure. Real-time PCR was performed by upeReal PerMix Plus (SYBRGreen) (TIANGEN) and the Applied Biosystems 7500 Real-time PCR System (Applied Biosystems, Inc., Carlsbad, CA, United States). Through the 2–11ΔΔCt method, the relative expressions of genes were calculated. Internal references were GAPDH. The primers of genes were summarized in Table S1.

### Statistical analysis

Statistical analyses were performed using R v.4.0.3. Multiple groups of continuous variables were analyzed using the chi-square test. Univariate, multivariate Cox regression and LASSO regression analyses were performed to evaluate survival. A *p* value of < 0.05 was considered statistically significant.

### Results

In the present study, we first hypothesized that ferroptosis-related genes could play a role in antibody-mediated immune rejection following renal allograft and that they could act as biomarkers for graft loss prediction. Next, we identified 22 DFGs between the graft rejection and non-rejection groups or between the graft failure and non-failure groups. Moreover, the results of Kaplan-Meier survival analysis suggested that all of them were closely related to graft loss in renal allograft patients. Finally, a prognostic ferroptosis-related gene signature based on six ferroptosis-related genes was constructed to predict graft loss following renal allograft using univariate Cox and Lasso Cox analyses. Furthermore, we investigated the independent predictive value of the gene signature and its related functions. Therefore, the present study might contribute to establishing a new strategy for the prediction of graft loss following renal allograft, increasing the understanding of the roles of ferroptosis in renal allografts.

### Screening of DFGs and functional annotation

**Figure 1.**
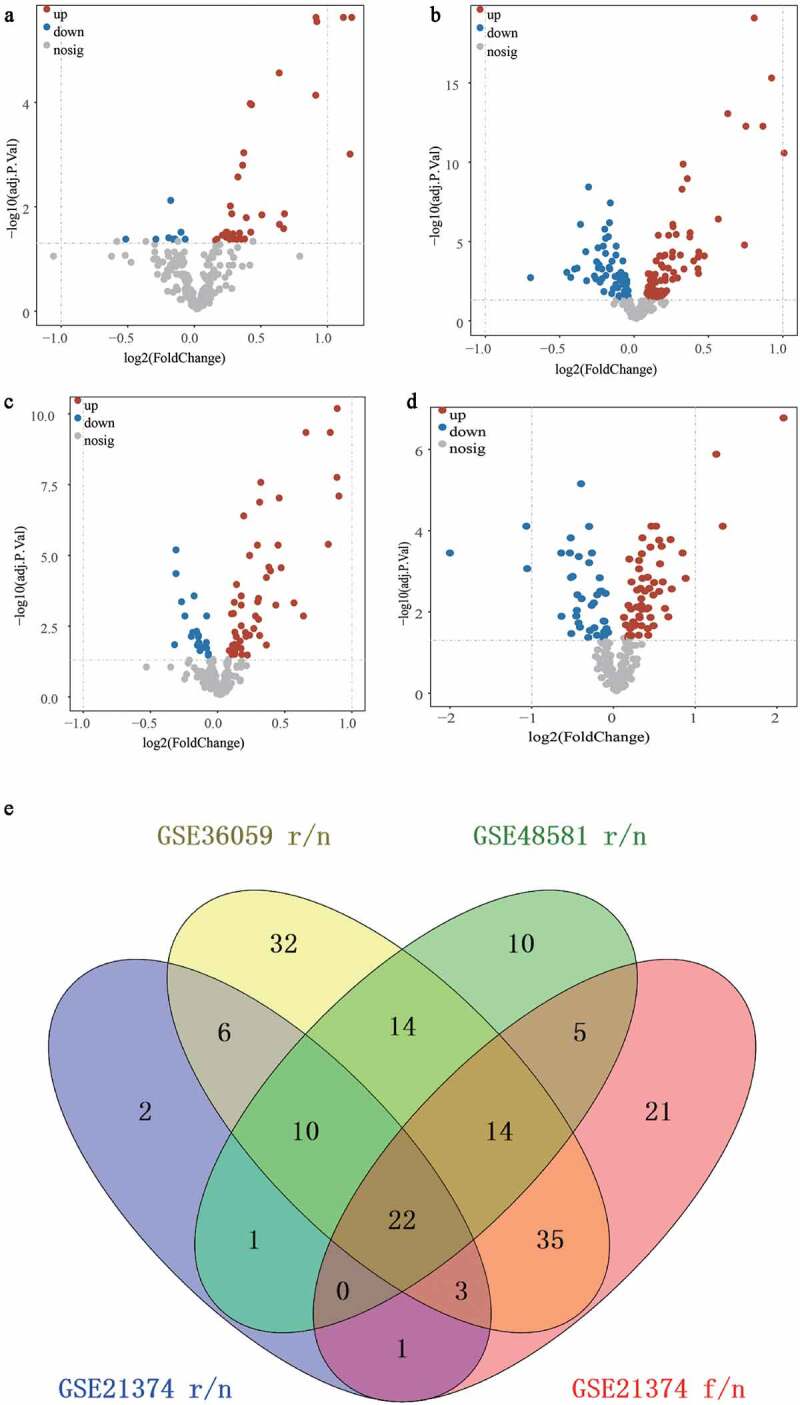
Identification of the DFGs related to rejection and graft loss. (a-c) The DFGs between rejection and non-rejection group in GSE21374 (a) GSE36059 (b) and GSE48581 (c), respectively. (d) The DFGs between the group which graft loss time less than survival medium time and the group which without graft loss and survival more than survival medium time in GSE21374 dataset. (e) The Venn diagram showed the overlapping genes of these DFGs

**Figure 2.**
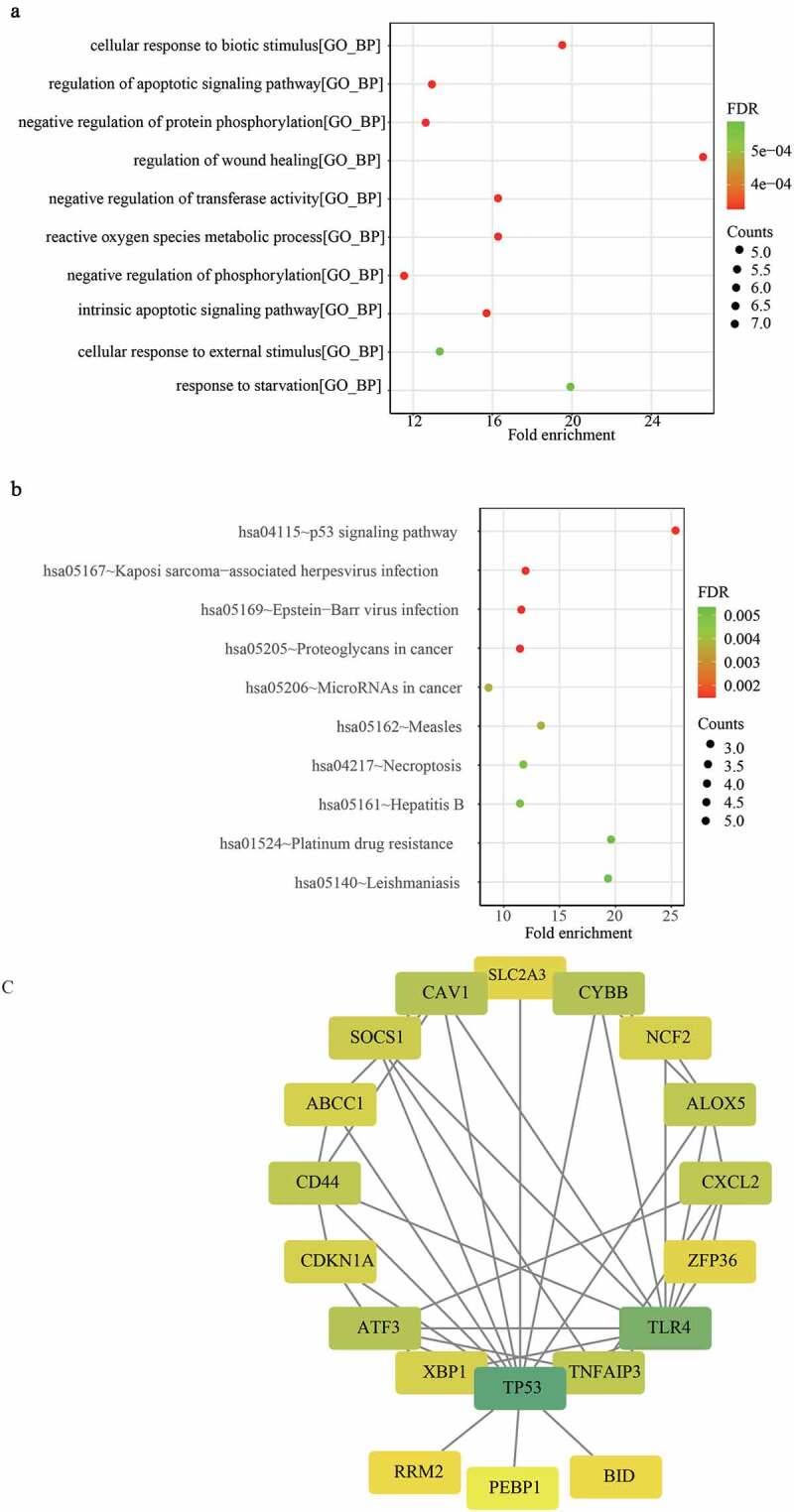
GO annotation and KEGG pathway enrichment analysis and PPI network construction. (a) The enriched top 10 biological processes by 22 DFGs. (b) The top 10 KEGG pathways enriched in by 22 DFGs. (c) The PPI network of the 22 DFGs

The three datasets included 244 ferroptosis genes. With the cutoff of adj.P.Val ≤ 0.05, we identified 37, 72, and 54 up-regulated, and 8, 64, and 22 down-regulated ferroptosis genes between the graft rejection and non-rejection groups in GSE21374 (Figure 1(a)), GSE36059 (Figure 1(b)) and GSE48581 (Figure 1(c)), respectively. We then acquired a total of 101 differentially expressed ferroptosis-related genes (DFGs) between the graft failed and non-failure groups in GSE21374 (Figure 1(d)). Finally, 22 DFGs were obtained using the R package ‘Venn diagram’ (Figure 1(e)). Moreover, the functional annotation of 22 DFGs showed that GO terms including negative regulation of protein phosphorylation, regulation of the apoptotic signaling pathway, negative regulation of transferase activity, and the intrinsic apoptotic signaling pathway, were activated (Figure 2(a)). KEGG terms, such as Kaposi sarcoma-associated herpesvirus infection, Epstein-Barr virus infection, the HIF-1 signaling pathway, the NOD-like receptor signaling pathway, autophagy, proteoglycans in cancer, and the p53 signaling pathway, were significant enriched (Figure 2(b). These results suggest that these signaling pathways may play key roles in renal allografts.

### Establishment of the PPI network

To explore the interactions of 22 DFGs at the protein level, we constructed a PPI network. After removing some loosely connected nodes and isolated nodes, an interaction network containing 19 genes and 44 interaction relationships was generated. The Cytoscape results showed that Tumor Protein (TP53) and Toll-like receptor 4 (TLR4) had the most nodes in the network (degrees > 10) (Figure 2(c)), suggesting that TP53 and TLR4 are the most important genes in the PPI network, and they may play an important role in renal allograft outcomes.

### Identification of graft loss-related DFGs

**Figure 3.**
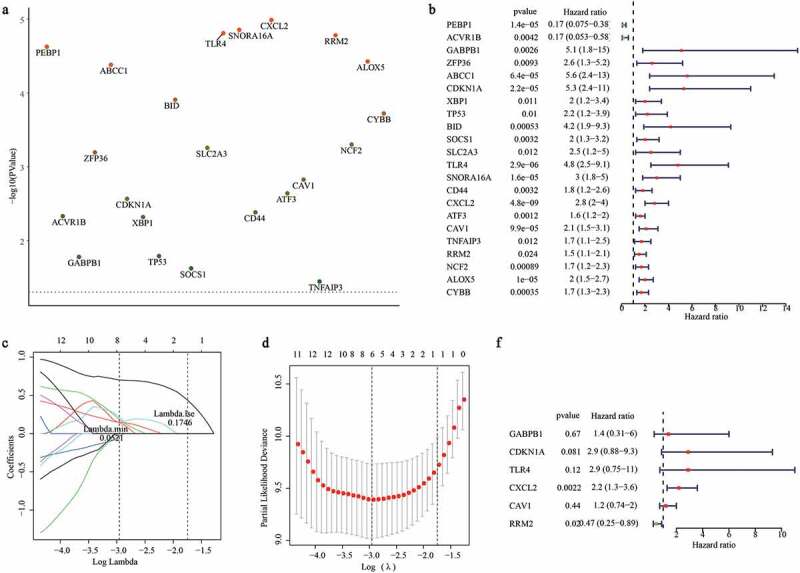
Construction of a ferroptosis related gene signature for predicting the graft loss of renal allograft. (a) K-M survival analysis showed that 22 DFGs were related to the graft loss. (b) univariate Cox regression analysis also showed 22 DFGs were related to the graft loss. (c) Lambda value of the 22 DFGs in LASSO model. (d) The optimal lambda value in Lasso analysis. (e) The optimal DFGs selected by multivariate Cox regression analysis

We performed Kaplan-Meier survival analysis based on the expression values of DFGs and graft loss information. After filtering for significant differences (*p* ≤ 0.05) (Figure 3(a)), we found that phosphatidylethanolamine binding protein 1 (PEBP1), activin a receptor type 1B (ACVR1B), GA-binding protein transcription factor subunit beta 1 (GABPB), ZFP36 ring finger protein (ZFP36), ATP binding cassette subfamily C member 1 (ABCC1), cyclin-dependent kinase inhibitor 1A (CDKN1A), X-Box binding protein 1 (XBP1), TP53, BH3 interacting domain death agonist (BID), suppressor of cytokine signaling 1 (SOCS1), solute carrier family 2 member 3 (SLC2A3), toll-like receptor 4 (TLR4), small nucleolar RNA, H/ACA Box 16A (SNORA16A), CD44 Molecule (CD44), C-X-C motif chemokine ligand 2 (CXCL2), activating transcription factor 3 (ATF3), caveolin 1 (CAV1), TNF alpha induced protein 3 (TNFAIP3), ribonucleotide reductase subunit M2 (RRM2), neutrophil cytosolic factor 2 (NCF2), arachidonate 5-lipoxygenase (ALOX5), and cytochrome B-245 beta chain (CYBB) were closely related to graft loss in renal allograft patients (Table 1).

**Table 1.** The results of Kaplan-Meier survival analysis for 22 DFGs

GENE	p value
AKR1C2	0.006096682
PEBP1	2.46E-05
ACVR1B	0.004833763
ATG3	0.518437442
ATG7	0.004347859
GABPB1	0.01726694
SLC40A1	0.716295856
ZFP36	0.000662971
SLC2A14	0.014497685
ABCC1	4.32E-05
CDKN1A	0.002822297
GCH1	0.500954384
XBP1	0.004991953
SLC2A6	0.088557098
TP53	0.016762405
MYB	0.192710539
BID	0.000128318
DDIT4	0.262393648
SOCS1	0.024709333
SLC2A3	0.000573896
TLR4	1.61E-05
SNORA16A	1.45E-05
IFNG	0.22360569
CD44	0.004294914
CXCL2	1.07E-05
ATF3	0.002380468
CAV1	0.00155036
TNFAIP3	0.03710298
RRM2	1.71E-05
NCF2	0.000518481
ALOX5	3.88E-05
CYBB	0.000196954

### Construction of the gene signature

As we confirmed that some DFGs were related to graft loss, we attempted to construct a gene signature to predict the outcomes for renal allograft patients. Firstly, the results of univariate Cox analysis showed that all DFGs were mostly related to graft loss (*p* < 0.05) (Figure 3(b)). Notably, among these genes, only PEBP1and ACVR1B could act as protective genes. Next, genes with a *p* value of < 0.05 were included in the Lasso Cox analysis. With the change in the penalty coefficient lambda, the coefficients of the variables were compressed to 0. When lambda was 0.0520857 (Figure 3(c, d)), GABPB1, CDKN1A, TLR4, CXCL2, CAV1, and RRM2 were selected to construct the gene signature using multivariate Cox regression analysis (Figure 3(e)).

### Assessment of the predictive value of the gene signature

**Figure 4.**
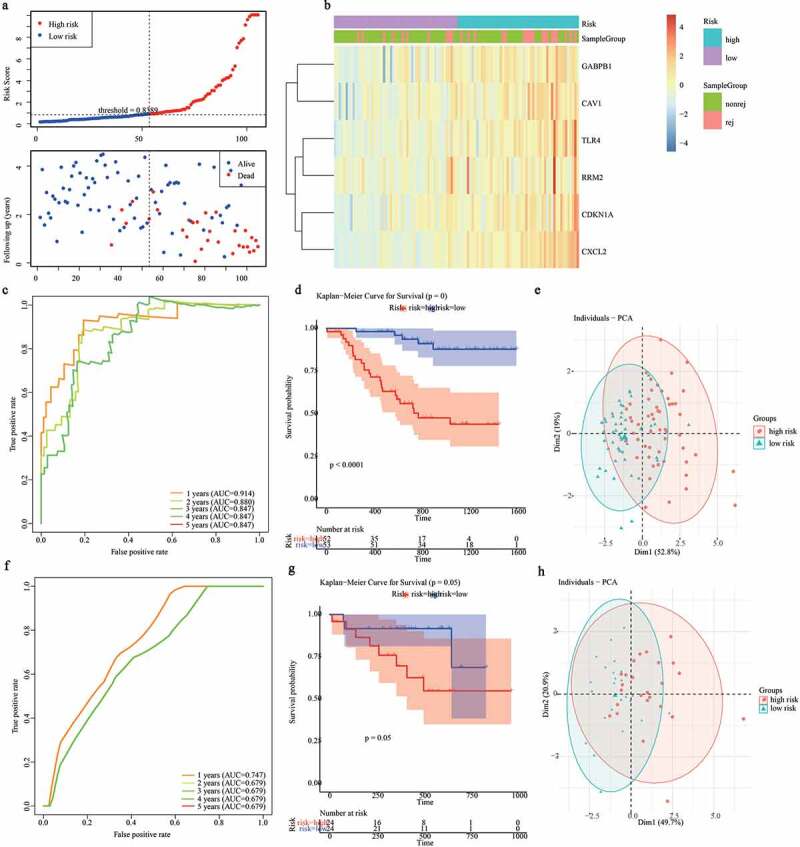
Evaluating the performance of ferroptosis related gene signature in the training set and validation set. (a) The distribution of risk scores and graft status in the training set. (b)The gene expression profiles of six genes in gene signature. (c, f) ROC curves presented the efficiency of the risk signature for predicting the graft loss in the training set (c) and the validation set (f). (d, g) The Kaplan-Meier survival curves showed the prognostic value of the gene signature in the training set (d) and validation set (g). (e, h) PCA analysis of the patients in high- and low-risk group in the training set (e) and validation set (h)

We assessed the predictive graft loss capability of the gene signature. We calculated the risk score for each patient based on the expression values and coefficients (Figure 4(a,b)). Then 1-, 2-, 3-, 4-, 5-year ROC curves were plotted and all areas under the ROC curves (AUCs) were greater than 0.8. The results indicated that the gene signature was an effective model for predicting graft loss (Figure 4(c)). Moreover, the time of graft loss between the high- and low-risk groups was significantly different (Figure 4(d)). Furthermore, the PCA results also showed a significant difference between the two groups (Figure 4(e)).

The validation set, which included 48 samples from the University of Minnesota in the GSE21374 dataset, was used to verify the applicability of the gene signature. Consistent with the results of the training set, the AUC value of the 3-year graft loss was 0.79, indicating the good performance of the gene signature (Figure 4(f)). Moreover, the high- and low-risk groups also presented a significant difference in survival probability and distribution (Figure 4(g,h)). Therefore, these results suggest that the gene signature had a good graft loss predictive value.

### Investigation of the independent predictive value of the gene signature

**Figure 5.**
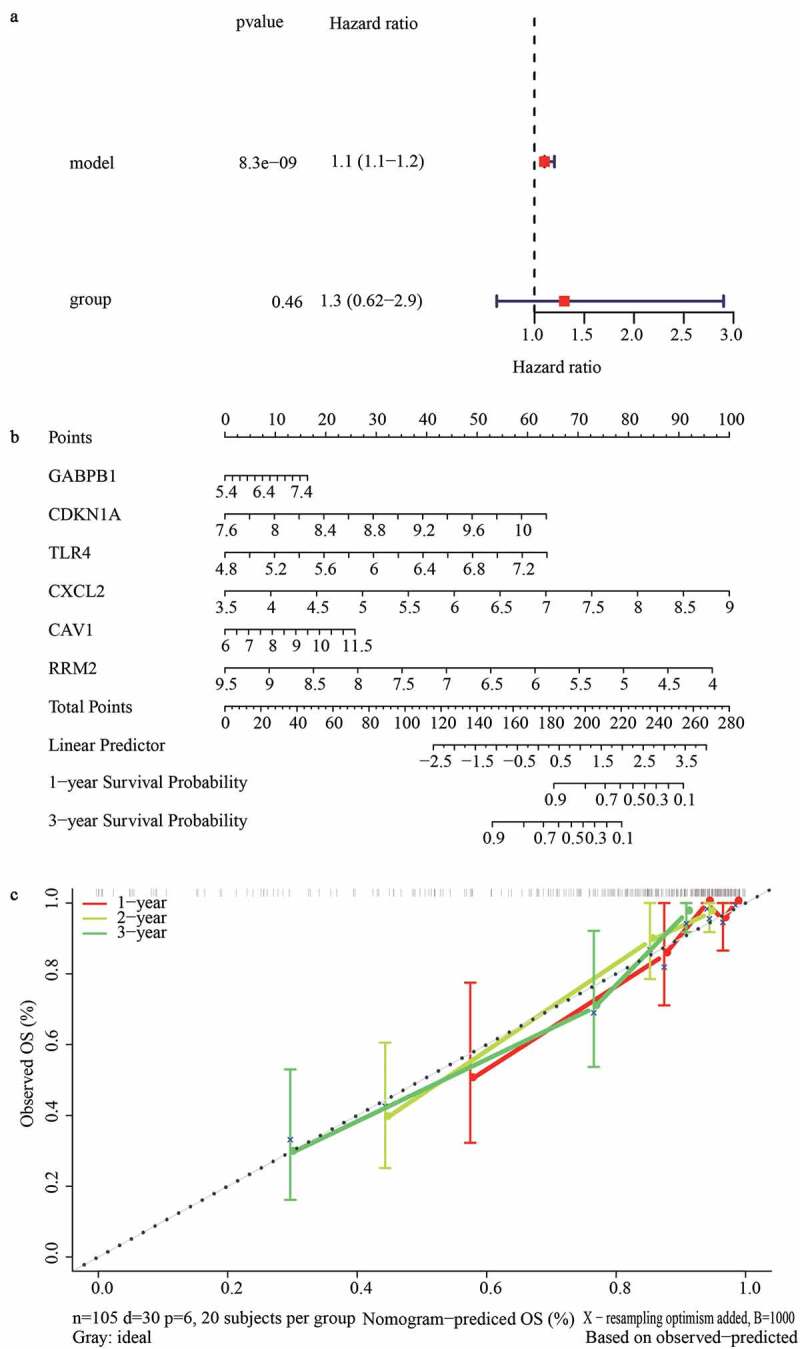
Construction of a nomogram for predicting the 1-, 2- and 3-year graft loss. (a) Univariate Cox regression analysis showed the contribution of each variable to the graft loss. (b) A nomogram for predicting 1-,2-and 3- graft loss. (c) The calibration plot presented the probability for predicting the 1-, 2- and 3- graft loss

To better utilize the gene signature, we first performed the univariate Cox regression analysis to determine whether the risk score and clinical features were independent prognostic predictors of graft loss. Surprisingly, we found that only the risk score was significantly correlated with graft loss in renal allograft patients (p < 0.05) (Figure 5(a)). Next, a nomogram was built to predict 1-, 2- and 3- year survival probability of renal allograft patients based on the risk signature genes using Cox regression analysis. In this nomogram, the total score was the sum of the expression values of each gene. The higher the score, the lower the median survival time and probability of graft loss (Figure 5(b)). In addition, the calibration plots showed that the nomogram had a better accuracy (Figure 5(c)). Furthermore, decision curve analysis (DCA) also showed that the clinical factor could not affect the benefits-decision of risk score (Figure 5(d)). Hence, these results indicate that we obtained a credible risk signature.

### GSEA

**Figure 6.**
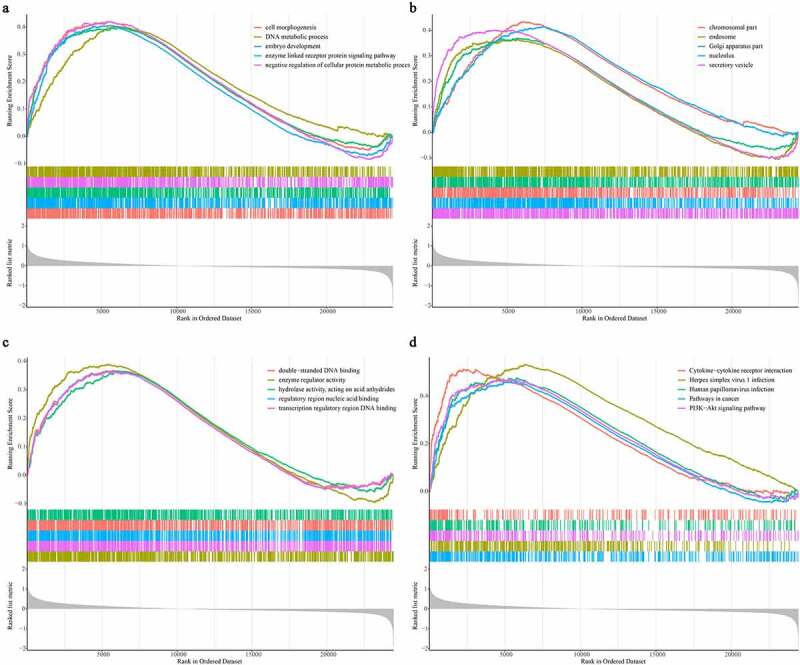
Identification of gene signature related function annotation. (a-d) The GO biological process terms (a), GO cellular components terms (b), GO molecular functions terms (c) and KEGG pathways (d) enriched by differentially expressed genes between high- and low-risk group

To explore the potential mechanisms underlying the gene signature in renal transplant patients, we screened differentially expressed genes in the high- and low-risk groups and found that a total of 108 genes (63 up-regulated and 45 down-regulated) were differentially expressed (Supplementary Figure S1). We then investigated the potential mechanism differentiating these two groups using GSEA. As shown in the Figure 6, GO biological process terms (Figure 6(a)), such as cell morphogenesis, DNA metabolic process, embryo development, and the enzyme-linked receptor protein signaling pathway, GO cellular component terms (Figure 6(b)) including the chromosomal part, endosome, Golgi apparatus, nucleolus, and secretory vesicle, as well as GO molecular function terms including double-stranded DNA binding, enzyme regulator activity, hydrolase activity acting on acid anhydrides, regulatory region nucleic acid binding, and transcription regulatory region DNA binding, were activated in the high-risk group (Figure 6(c)). Moreover, KEGG pathways, such as cytokine-cytokine receptor interaction, herpes simplex virus 1 infection, human papillomavirus infection, pathways in cancer, and the PI3K-Akt signaling pathway, were enriched in the high-risk group (Figure 6(d)).

### Quantitative Real-Time-PCR Validation

**Figure 7.**
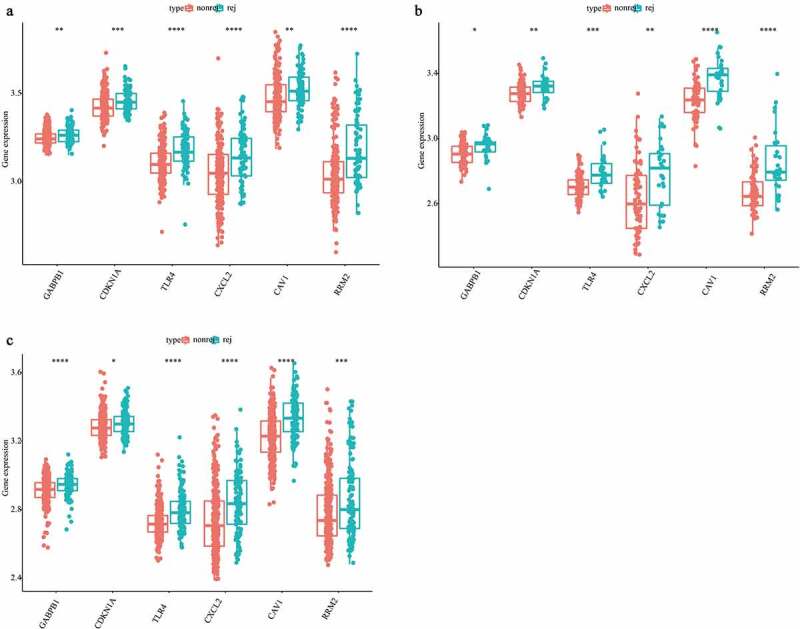
The expression levels of GABPB1, CDKN1A, TLR4, CXCL2, CAV1, and RRM2 between between graft rejection and non-rejection samples from the GEO database. (a) GSE48581. (b) GSE21374. (c) GSE36059

**Figure 8.**
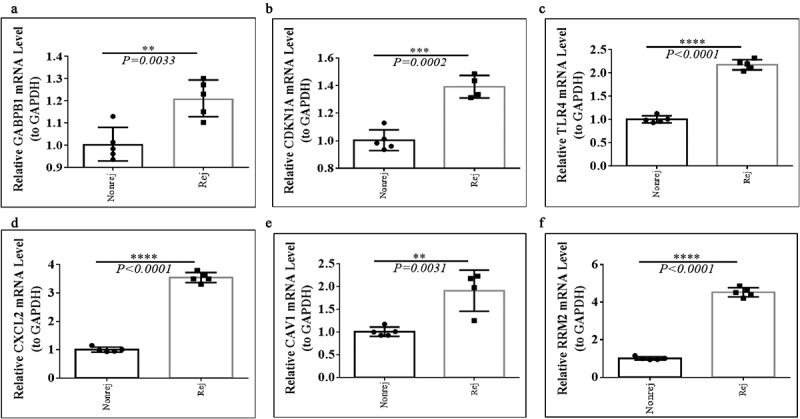
The expression levels of GABPB1, CDKN1A, TLR4, CXCL2, CAV1, and RRM2 between between graft rejection and non-rejection samples from Clinical PBMCs. (a) GABPB1. (b) CDKN1A. (c) TLR4. (d) CXCL2. (e) CAV1. (f) RRM2

For further investigating the expression levels of GABPB1, CDKN1A, TLR4, CXCL2, CAV1, and RRM2 between graft rejection and non-rejection, we performed Quantitative Real-Time-PCR validation. Notably, all the expression levels of GABPB1, CDKN1A, TLR4, CXCL2, CAV1, and RRM2 were up-regulated in rejection samples compared to non-rejection samples in three GEO database (Figure 7(a-c)). Consistent with the result of GEO database, we also found that the expression levels of GABPB1, CDKN1A, TLR4, CXCL2, CAV1, and RRM2 were up-regulated in rejection PBMCs compared to non-rejection PBMCs (Figure 8). Thus, GABPB1, CDKN1A, TLR4, CXCL2, CAV1, and RRM2 may play key roles in renal allograft.

## Discussion

Renal allograft is the most effective renal replacement therapy for patients with end-stage renal disease [1]. However, acute and chronic graft rejection greatly limits the efficiency of renal allografts [35,36]. Unfortunately, current diagnosis of graft rejections mainly relies on the pathology biopsy reports, which might lead to misdiagnosis due to considerable inter-observer disagreements [37,38]. Moreover, the molecular mechanism underlying graft rejection and loss is still not fully understood. Therefore, we hypothesized that rejection-related genes might act as biomarkers for predicting renal allograft loss. Emerging evidence indicates that ferroptosis is related to acute kidney injury and I/R injury [17,18,20]. However, the role of ferroptosis in renal allografts remains unclear. Hence, the present study aimed to investigate the role of ferroptosis in graft rejection and loss after renal allograft.

We first identified 22 DFGs between the rejection samples and non-rejection samples in three datasets from the GEO database. Next, the results of GO annotation suggested that 22 DFGs were mainly involved in the negative regulation of protein phosphorylation, regulation of the apoptotic signaling pathway, and the negative regulation of transferase activity (Figure 2(a)). Moreover, the results of the KEGG pathway analysis suggested that 22 DFGs were mainly related to Kaposi sarcoma-associated herpesvirus infection, Epstein-Barr virus infection, the HIF-1 signaling pathway, the NOD-like receptor signaling pathway, autophagy, proteoglycans in cancer, and the p53 signaling pathway (Figure 2(b)). Furthermore, 22 DFGs were associated with graft loss (Table 1). Finally, a ferroptosis-related gene signature including GABPB1, CDKN1A, TLR4, CXCL2, CAV1, and RRM2 was constructed to predict the graft loss following the renal allograft, which showed good performance in predicting graft loss. On the other hand, we also analyzed the GO functions and KEGG pathways associated with the gene signature and found that cytokine-cytokine receptor interaction, herpes simplex virus 1 infection, human papillomavirus infection, pathways in cancer, and the PI3K-Akt signaling pathway were mainly enriched in the high-risk group. Notably, we found that graft rejection was not associated with graft loss (Figure 5(a)), which might indicate that rejection is not an independent prognostic factor. Thus, we speculated that graft loss may rely on other factors. However, the results might be influenced by the number of samples. Hence, further studies are necessary to elucidate the association between graft rejection and loss.

GABPB1, has been associated with ferroptosis and can be used as a therapeutic target in hepatocellular carcinoma [39]. Moreover, GABPA regulates the expression of CDKN1A and serves as a tumor suppressor in bladder cancer [40]. However, the association between GABPA and renal allografts has not been reported. Thus, we speculated that GABPA might be related to graft loss by regulating ferroptosis. However, additional studies are needed to elucidate the role of GABPA in graft loss. CDKN1A, also known as p21, has been regarded as a key mediator of p53-dependent cell cycle arrest after DNA damage [41]. In addition, it has been suggested that CDKN1A can inhibit ferroptosis by inducing the TP53 signaling pathway [42]. Notably, we also found that TP53 was associated with patient survival (Figure 3(b)). Moreover, a recent study reported that CDKN1A participates in the pathogenesis of diabetic glomerular hypertrophy [43]. CDKN1A was also found to prevent renal interstitial fibrosis in diabetic nephropathy by inhibiting the expression of miR-93-5p [44]. Interestingly, another study also suggested that knocking out the CDKN1A gene can clearly improve chronic kidney disease [45]. Furthermore, CDKN1A affects ischemia-induced acute renal failure [46]. Thus, CDKN1A might play an important role in graft loss following renal allograft.

TLR4 has been suggested to act as mediators inflammatory mediators in the kidney. It has been shown that TLR4 was involved in renal fibrosis by mediating pro-inflammatory and pro-fibrotic pathways [47]. Similarly, TLR4 is also associated with inflammation in renal I/R injury [48]. Importantly, TLR4 is involved in the activation of immune and inflammatory responses [49]. Thus, we speculated that TLR4 may affect graft loss. CXCL2, an ELR-CXC chemokines, has been suggested to be related to uric acid nephropathy [50]. In addition, CXCL2 is also involved in renal I/R injury [51] and sepsis-associated acute kidney injury [52]. Therefore, our study further revealed that CXCL2 is also related to graft loss in renal allografts.

Regarding the remaining two genes, it has been suggested that CAV1 plays an important role in diabetic nephropathy [53,54]. More importantly, the genotype of CAV1 can affect the renal transplant outcome, which is consistent with our results [55]. On the other hand, Hence, a recent study suggested that CAV1 can be used as a biomarker to distinguish renal allograft tolerance from chronic antibody-mediated rejection [56]. Thus, our study further revealed that CAV1 was related to rejection and graft loss following the renal allografts. To our knowledge, there has been no report on the role of RRM2 in several kidney diseases, except in renal carcinoma [57,58]. Therefore, it is urgent to further explore the roles of RRM2 in renal allograft and other non-cancerous kidney diseases.

In summary, ferroptosis-related genes, including GABPB1, CDKN1A, TLR4, CXCL2, CAV1, and RRM2, might be used to predict graft loss following renal allograft. However, the mechanisms of action and the correlation between graft rejection and loss remain unclear.

## Conclusion

In conclusion, we established a ferroptosis-related gene signature, including GABPB1, CDKN1A, TLR4, CXCL2, CAV1, and RRM2, to predict graft loss following allogeneic kidney transplantation (renal allograft) based on graft rejection-related genes. In addition, we developed a nomogram for better prediction of graft loss in renal allografts. Thus, GABPB1, CDKN1A, TLR4, CXCL2, CAV1, and RRM2 may act as biomarkers of graft loss after renal allograft. Therefore, these findings may contribute to increasing the understanding of the role of ferroptosis in renal allografts and improve the prediction of graft loss following renal allografts. However, the mechanisms underlying the roles of GABPB1, CDKN1A, TLR4, CXCL2, CAV1, and RRM2 in renal allografts remain unclear. Thus, additional research is needed to elucidate these mechanisms and further explore the correlation between graft rejection and loss.

## Supplementary Material

Supplemental Material

Supplemental Material

## Data Availability

The datasets (GSE21374, GSE36059, and GSE48581) included in the present study can be found in GEO database (https://www.ncbi.nlm.nih.gov/geo/).

## Highlights

1. A ferroptosis related gene signature for predicting the graft loss after renal allograft was established
2. The gene signature could act as a an independent factor
3. The immune-related pathways and cytokine signaling pathways were mostly enriched in the high-risk group
