# Supplementary material for: A ferroptosis-related gene signature for graft loss prediction following renal allograft
Source: Bioengineered. 2021 Aug 1;12(1):4217–32. doi: 10.1080/21655979.2021.1953310 (PMC8806795; doi:10.1080/21655979.2021.1953310)
Supplement: Supplemental Material [file KBIE_A_1953310_SM0652.docx]

Table S1 The primer sequences and annealing temperatures of GABPB1, CDKN1A, TLR4, CXCL2, CAV1, and RRM2

| Gene | Sequence（5^，^→3^,^） | | Length | Tm | GC% |
| --- | --- | --- | --- | --- | --- |
| GABPB1 | Forward Primer | TGCCAGATGGACAACAAGTATT | 22 | 57.97 | 40.91 |
|  | Reverse Primer | GCAAAGCACACCGGGTAAAA | 20 | 59.61 | 50.00 |
| CDKN1A | Forward Primer | GTCCAGCATGCTCCAGATTTC | 21 | 59.33 | 52.38 |
|  | Reverse Primer | GATGTAGAGCGGGCCTTTGA | 20 | 59.82 | 55.00 |
| TLR4 | Forward Primer | CCGTTTTATCACGGAGGTGGT | 21 | 60.34 | 52.38 |
|  | Reverse Primer | CTGCCTAAATGCCTCAGGGG | 20 | 60.47 | 60.00 |
| CAV1 | Forward Primer | CTGTCGGAGCGGGACATC | 18 | 59.89 | 66.67 |
|  | Reverse Primer | TGTTTAGGGTCGCGGTTGAC | 20 | 60.60 | 55.00 |
| CXCL2 | Forward Primer | CTTGCCAGCTCTCCTCCTC | 19 | 59.48 | 63.16 |
|  | Reverse Primer | AGGGGCGCTCCTGCT | 15 | 59.92 | 73.33 |
| RRM2 | Forward Primer | ACTATGCTCTCCCTCCGTGT | 20 | 60.03 | 55.00 |
|  | Reverse Primer | GCTGCTTTAGTTTTCGGCTCC | 21 | 60.14 | 52.38 |
