# Supplementary figures and images for: A ferroptosis-related gene signature for graft loss prediction following renal allograft
Source: Bioengineered. 2021 Aug 1;12(1):4217–32. doi: 10.1080/21655979.2021.1953310 (PMC8806795; doi:10.1080/21655979.2021.1953310)

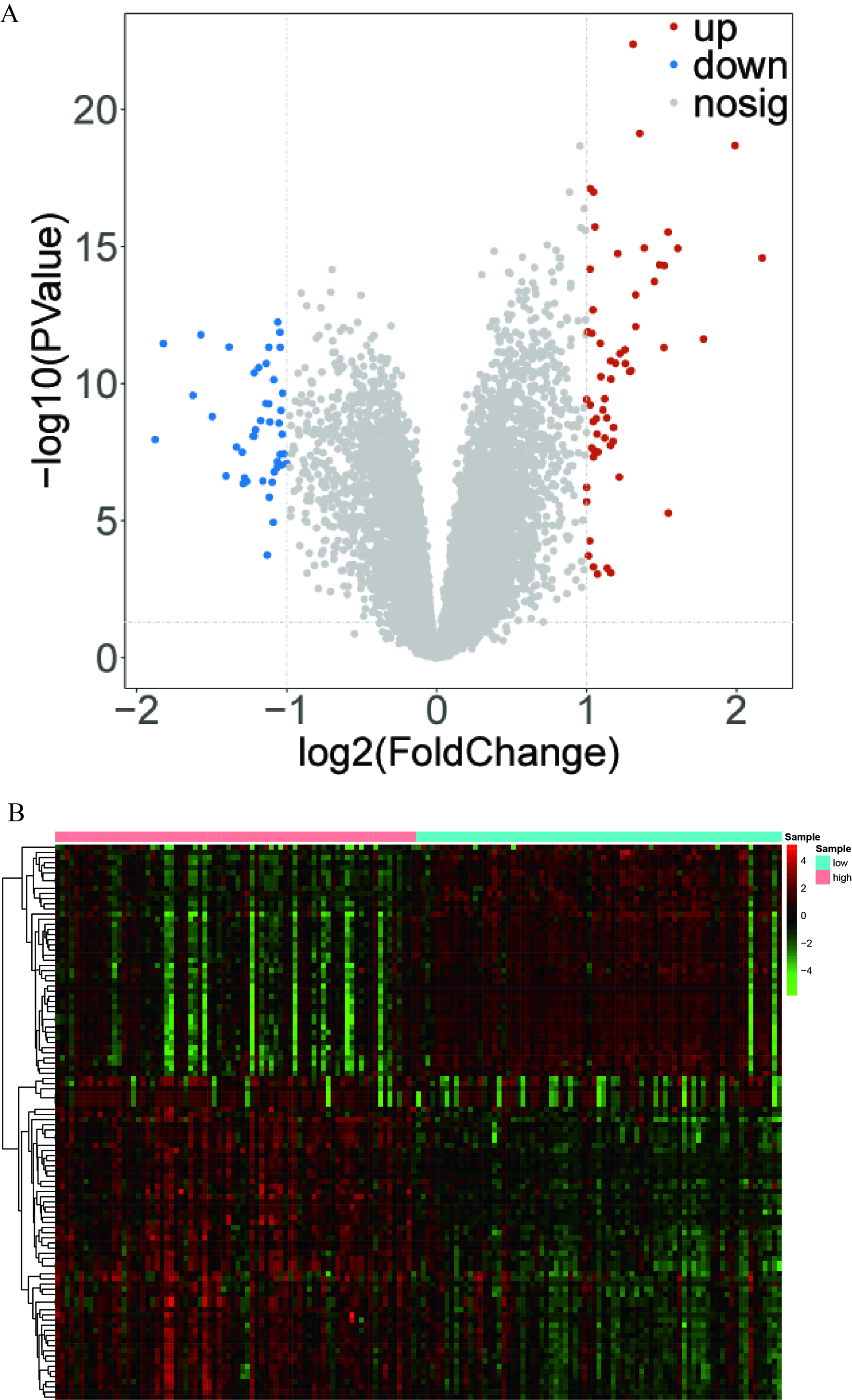

Supplement: Supplemental Material [file KBIE_A_1953310_SM0574.tif]
